# Dynamic Viscoelastic Behavior of Maize Kernel: Application of Frequency–Temperature Superposition

**DOI:** 10.3390/foods13070976

**Published:** 2024-03-22

**Authors:** Shaoyang Sheng, Min Wu, Weiqiao Lv

**Affiliations:** 1 School of Public Health, Anhui Medical University, Hefei 230032, China; 2 College of Engineering, China Agricultural University, Beijing 100083, China

**Keywords:** maize kernel, frequency sweep test, frequency–temperature superposition, modified Cole–Cole analysis

## Abstract

Maize kernels were treated using two varieties of drying methodologies, namely combined hot air- and vacuum-drying (HAVD) and natural drying (ND). We performed frequency sweep tests, modified Cole–Cole (MCC) analysis, and frequency–temperature superposition (FTS) on these kernels. The kernels’ elastic and viscous properties for ND were higher than those for HAVD. The heterogeneous nature of maize kernel may account for the curvature in MCC plot for the kernel treated by HAVD 75 °C and the failure of FTS. MCC analysis was more sensitive than FTS. The kernel treated by HAVD 75 °C demonstrated thermorheologically simple behavior across the entire temperature range (30–45 °C) in both MCC analysis and FTS. The frequency scale for the kernel treated using HAVD 75 °C was broadened by up to 70,000 Hz. The relaxation processes in the kernel treated by HAVD 75 °C were determined to be mainly associated with subunits of molecules or molecular strands. The data herein could be utilized for maize storage and processing.

## 1. Introduction

Maize is among the most important crops in the world [1]. It is generally harvested with high moisture content, and it should be dried before storage.

The frequency sweep test measures the system response to a constant stress amplitude. This method reveals the loss modulus (viscous response) and the storage modulus (elastic response) [2]. Li et al. [3] investigated the frequency dependence of sweet potato. The authors reported that resonance appeared in both compression and three-point bending modes. Ditudompo et al. [4] reported that the loss and storage moduli of extruded cornstarch showed an increasing trend with an increase in frequency. Shi et al. [5] investigated the frequency dependence of starch films. These scholars reported that the films demonstrated dominant elastic behavior. Ozturk and Takhar [6] studied the influence of drying duration on the viscoelasticity of strawberries, reporting that the loss and storage modulus values decreased with the rise in drying duration.

Increasing the frequency has a similar effect as decreasing the temperature on the viscoelastic behavior of material. This is called frequency–temperature superposition (FTS), and may be viewed as a type of time–temperature superposition (TTS). Commercial instruments’ measurement frequency range is restricted to a limited range. FTS can be used to resolve this problem, being employed to extend the frequency range by superposing isothermal frequency (ITF) curves to generate a master curve. If a viscoelastic material follows FTS, it is considered thermorheologically simple; if it does not follow, it is regarded as thermorheologically complex. Ahmed [7] tested the applicability of FTS for mung bean starch dispersions and reported that the storage modulus superposition was found to fail in the temperature range of 70–95 °C. Altay and Gunasekaran [8] successfully applied FTS to a system of gelatin–xanthan gum containing sucrose and glucose syrup. Razavi et al. [9] reported that the sage seed gum–xanthan gum mixture demonstrated thermorheologically simple behavior at a ratio of 3:1 and 1:0.

Modified Cole–Cole (MCC) analysis is another solution for the problem of the limited measurement frequency range of commercial instruments. For the MCC analysis, ITF data can be superposed without data manipulation compared with FTS [10]. When investigating thermorheologically complex and simple materials, MCC analysis’s failure and success for superpossibility is similar to FTS. Meza et al. [11] compared frozen and refrigerated cheeses for the MCC analysis, reporting that storage and loss moduli could be superposed in the range of 10–30 °C. Nevertheless, at temperatures above 30 °C, the moduli failed to superimpose. Ahmed et al. [12] reported that temperature did not exert a remarkable impact on the MCC plot of poly(ε-caprolactone) nanocomposites containing 10% clay. Wu et al. [13] employed MCC analysis to reveal the microstructure homogeneity and isotropy of polysaccharide from *Sophora alopecuroides* L. seeds. They reported that the results suggested the structural micro-heterogeneity of polysaccharide from *Sophora alopecuroides* L. seeds to some extent.

To our knowledge, no research has been published on the application of FTS for maize kernel. As such, the goals of this study were (i) to employ FTS and MCC analysis on maize kernel and (ii) to predict the viscoelasticity of maize kernels in a broad frequency scale by utilizing FTS.

## 2. Materials and Methods

### 2.1. Materials

The Nongda 86 maize species was utilized in our work and its seed kernels were supplied by Beijing Sinong Seed Company Limited (Beijing, China). The type of the kernel was half horse-toothed. The kernel’s dimensions were approximately 4 mm in thickness, 8 mm in width, and 10 mm in length. Before usage, these kernels were sealed in plastic bags and stored at 4 °C. Their moisture content was 27%.

### 2.2. Drying

Two methods were employed to dry the maize kernels, as detailed below. A Shizuoka Seiki GTR800E Single Kernel Moisture Tester (Shizuoka, Japan) was employed to determine the kernels’ moisture content.

#### 2.2.1. Combined Hot Air- and Vacuum-Drying (HAVD)

A two-stage drying approach was employed in our work, involving hot air-drying followed by vacuum-drying. The kernels were first dried in a Shanghai Yiheng Scientific Instrument hot air oven (Shanghai, China) at 50 °C until reaching 18% moisture content. Then, the efficiency of drying began to decrease. This occurred because the kernel’s dry surface plays the role of a diffusion barrier, which reduces the rate of moisture transfer [14].

In the second drying stage, the kernels were dried in a −0.1 MPa Taisite Instrument vacuum drier (DZ-3, Tianjin, China) at various temperatures (35 °C, 45 °C, 55 °C, 65 °C, and 75 °C) down to 13% moisture content. Finally, the kernels were stored in ziplock bags in a 4 °C freezer.

#### 2.2.2. Natural Drying (ND)

The kernels were naturally dried in a chamber until a moisture content of 13% was reached. Then, the kernels were stored in ziplock bags in a 4 °C freezer.

### 2.3. Sample Preparation

A kernel sample contained the following components: crude starch (about 70%), crude protein (about 11%), and crude fat (about 4%). The sample preparation was conducted according to our previous investigation [15]. Each maize kernel was carefully rubbed with sandpaper to make its surface flat. A PRO-MAX electronic caliper (Fowler, Newton, MA, USA) with 0.01 mm precision was utilized to measure each kernel’s dimensions.

### 2.4. Frequency Dependency

Frequency sweep experiments were conducted in compression mode, utilizing a TA Instruments Q800 dynamic mechanical analyzer (DMA, New Castle, DE, USA). Every kernel was placed between the bottom and top parallel plates of the DMA with the germ side facing down. A preload of 0.1 N was supplied to guarantee proper contact between the compression plate and the kernel’s surface. The frequency sweep (1–30 Hz) experiments were conducted at 0.35% strain at four temperatures (45 °C, 40 °C, 35 °C, and 30 °C) and the ITF data were acquired.

Power law equations (Equations (1) and (2)) were employed to describe the frequency dependence of *E*′ and *E*″ [16]:

(1) $$E^{\prime }=K^{\prime }\cdot f^{n^{\prime }}$$

(2) $$E^{\prime \prime }=K^{\prime \prime }\cdot f^{n^{\prime \prime }}$$

where *f* (Hz) denotes the frequency; *E*′ (MPa) and *E*″ (MPa) represent the storage modulus and loss modulus, respectively; *n*′ (dimensionless) and *n*″ (dimensionless) denote the frequency exponents; and *K*′ (MPa·s^n^) and *K*″ (MPa·s^n^) denote the proportionality constants and reflect the elasticity and viscous property, respectively.

### 2.5. Frequency–Temperature Superposition

FTS was utilized to predict the *E*′ and *E*″ values of maize kernels in a wider frequency range. By shifting the ITF data horizontally, the master curves were produced. The horizontal shift factor *α_T_* can be obtained utilizing the Arrhenius model.

(3) $$\log\alpha_{T}=\frac{E_{a}}{2.303R}\left(\frac{1}{T}-\frac{1}{T_{0}}\right)$$

where *T* (K) denotes the experimental temperature, *T*_0_ (K) denotes the reference temperature, *R* (J/(K·mol)) denotes the gas law constant, and *E_a_* (kJ/mol) denotes the activation energy. Regression analysis was performed using Equation (3) to obtain the coefficient of determination (*R*^2^) and *E_a_*. As a rule, the choice of the reference temperature can be arbitrary or according to the temperature of interest. In this instance, a temperature of 45 °C was utilized as *T*_0_.

### 2.6. Statistical Analysis

All DMA measurements were conducted in triplicate. The DMA data were collected utilizing TA Instruments Universal Analysis 2000 software version 4.3A (New Castle, DE, USA). Nonlinear regression analysis was performed utilizing SPSS 17.0 (SPSS Inc., Chicago, IL, USA). TA Instruments Rheology Advantage Data Analysis software version 5.7.0 (New Castle, DE, USA) was utilized to perform FTS.

## 3. Results and Discussion

### 3.1. Frequency Dependency

**Figure 1:**
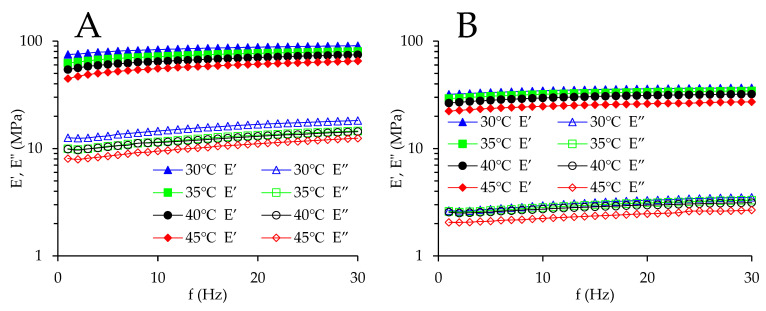

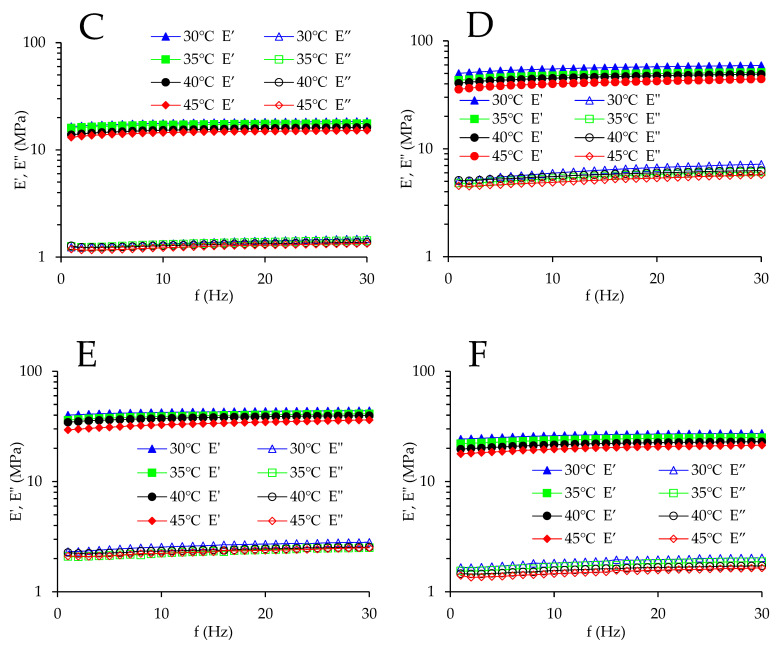
Storage modulus (*E*′) and loss modulus (*E*″) against frequency curves of maize kernels dried utilizing various drying treatments at different temperatures. (**A**) natural drying (ND); (**B**) hot air-drying 50 °C + vacuum-drying 35 °C (HAVD 35 °C); (**C**) hot air-drying 50 °C + vacuum-drying 45 °C (HAVD 45 °C); (**D**) hot air-drying 50 °C + vacuum-drying 55 °C (HAVD 55 °C); (**E**) hot air-drying 50 °C + vacuum-drying 65 °C (HAVD 65 °C); (**F**) hot air-drying 50 °C + vacuum-drying 75 °C (HAVD 75 °C).

Figure 1 presents *E*′ and *E*″ against frequency curves for maize kernels treated using different drying conditions at various temperatures. The curves show similar trends regardless of the drying condition. When the frequency increases, both *E*′ and *E*″ rise for all kernels. The explanation behind this is as follows: the kernels have time to relax at low frequencies and show relatively soft and low moduli; conversely, at high frequencies, the kernels have insufficient time to relax and display relatively hard and high moduli [3]. A similar phenomenon was reported for potato flour [17]. Such behavior implies that at high frequencies, more mechanical energy is stored inside the kernel, while at low frequencies, more energy is released. Furthermore, *E*′ is much higher than *E*″ in the whole frequency range. This suggests that the kernels treated by ND and HAVD both show dominant elastic characteristics rather than viscous characteristic, and the samples show solid-like behavior [18]. As shown in Figure 1, as the frequency increases, *E*′ and *E*″ increase very slowly. The weak frequency dependency of *E*′ and *E*″ indicates that these kernels possess glassy consistencies [19]. Moreover, *E*′ decreases with the temperature. This is because the molecular motion increases as the temperature rises, and thus the kernel becomes flexible and soft [20]. *E*″ also decreases with the temperature. The reason for this is as follows: as the temperature increases, the molecular spacing is elevated, the free volume between molecules becomes larger, the friction between chain segments decreases under dynamic compressive force, and *E*″ falls. The decrease in storage modulus and loss modulus with elevated temperature was also reported in polysaccharide from *Sophora alopecuroides* L. seeds [13]. Figure 1 also shows that *E*′ and *E*″ for HAVD are lower than those for ND. The decrease in *E*′ and *E*″ illustrates that maize kernel’s elastic and viscous characteristics for HAVD are lower than those for ND. The reason for this is described as follows: for HAVD, because the temperature of hot air-drying is relatively high, the moisture on the kernel surface evaporates rapidly, while the diffusion of moisture from inside the kernel to the kernel surface is relatively slow. As a result, a moisture gradient is built up. This causes internal stress, leading to crack, which causes a decrease in kernel mechanical strength. Therefore, *E*′ and *E*″ values attained for HAVD are lower than those for ND.

Table 1 displays the power law model parameters for maize kernels dried utilizing different drying treatments. *R*^2^ ranges from 0.963 to 0.996 for *E*′ and from 0.769 to 0.956 for *E*″, indicating that the power law equation is capable of fitting the *E’* and *E*″ data. For all kernels, *K*′ is higher than *K*″ (at the same temperature). This confirms the low values of tan *δ*. *K*′ and *K*″ for HAVD are lower than those for ND (at the same temperature). This is also parallel to the results shown in Figure 1. Furthermore, *K*′ and *K*″ for HAVD display fluctuations with drying conditions and do not demonstrate an increasing or decreasing trend (at the same temperature). A similar phenomenon is observed in *n*′ and *n*″ for HAVD. This may be due to the sample-to-sample variation in maize kernel. No two kernels are exactly the same. The germ plays a large role in kernel structure. Even when two kernels are similar in shape, the position and size of germ within every kernel are different. Sampling kernels from a specific position on the cob has the potential to minimize variation. Extensive test replications may also be needed [20]. According to Table 1, *n*′ and *n*″ for HAVD are lower than those for ND (at the same temperature), suggesting that the frequency sensitivity of kernel treated using HAVD is lower than that of kernels treated with ND [21]. Moreover, *n*′ and *n*″ are close to 0. Similar frequency exponent values were reported for starch film [22]. For maize kernels, when the temperature increases from 30 °C to 45 °C, *n*′ increases (except for the following cases: HAVD 55 °C at 30 °C and HAVD 45 °C at 35 °C and 45 °C), showing increasing frequency sensitivity. In addition, for the same treatment, *n*′ is lower than *n*″ (at the same temperature), except for in the following cases: HAVD 45 °C at 40 °C and HAVD 65 °C at 45 °C. This indicates that the frequency dependence of *E*″ is higher than that of *E’*.

### 3.2. Frequency–Temperature Superposition

**Figure 2:**
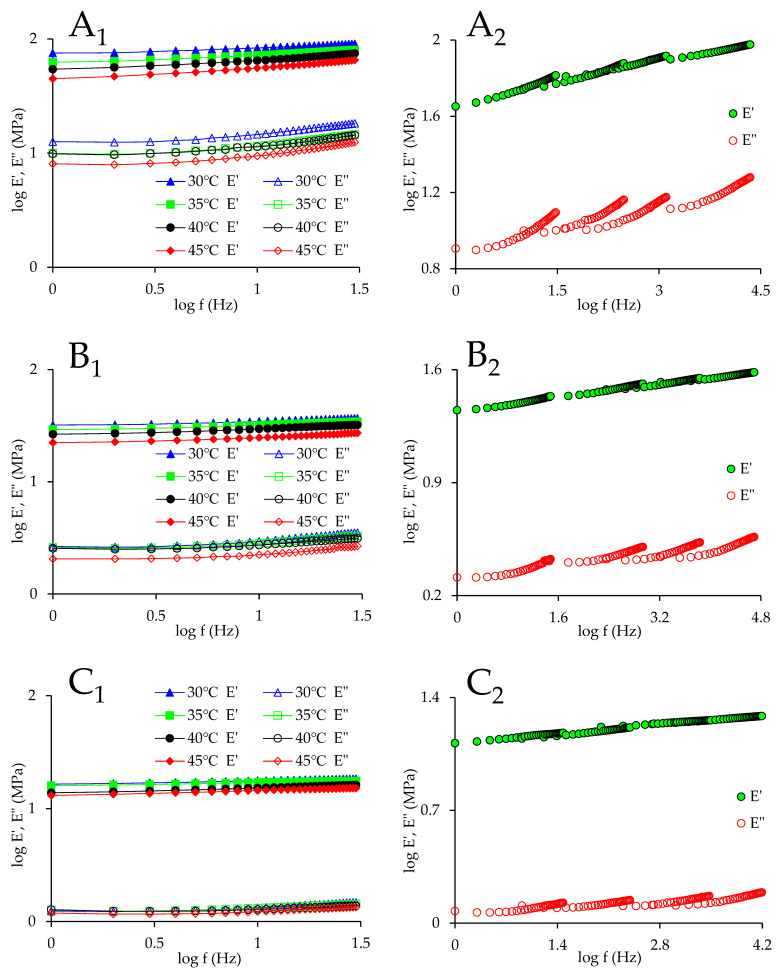

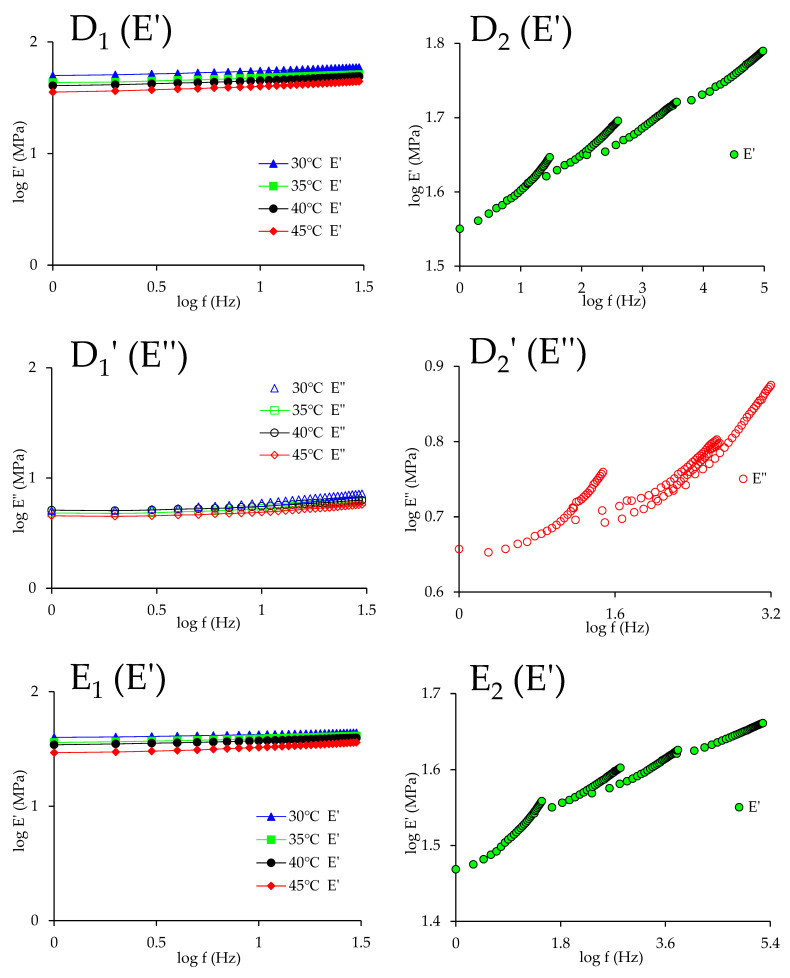

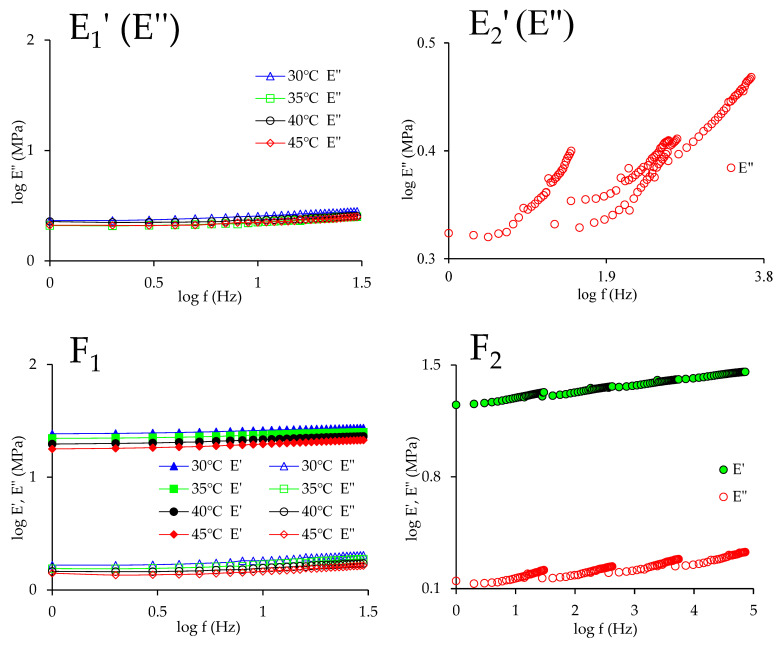
Left panels represent the *E*′ and/or *E*″ against frequency curves for kernel samples dried utilizing various drying treatments at different temperatures, and the right panels represent the master curves. (**A_1_**,**A_2_**)—ND; (**B_1_**,**B_2_**)—HAVD 35 °C; (**C_1_**,**C_2_**)—HAVD 45 °C; (**D_1_** (*E*′), **D_2_** (*E*′))—HAVD 55 °C; (**D_1_′** (*E*″), **D_2_′** (*E*″))—HAVD 55 °C; (**E_1_** (*E*′),**E_2_** (*E*′))—HAVD 65 °C; (**E_1_′** (*E*″),**E_2_′** (*E*″))—HAVD 65 °C; (**F_1_**,**F_2_**)—HAVD 75 °C.

The applicability criteria for FTS are listed as follows [23]: (i) neighboring curve shapes must match precisely; (ii) the same *α_T_* should be applicable to all viscoelastic parameters; and (iii) the relationship between temperature and *α_T_* should satisfy the empirical models. Figure 1A–F were replotted in log–log scale (left panels in Figure 2) in order to employ FTS. By shifting the frequency sweep curves (left panels in Figure 2) horizontally, the master curves (right panels in Figure 2) were produced. Table 2 shows the horizontal shift factor *α_T_* for maize kernels treated using different drying conditions. Table 3 shows *E_a_* acquired via the utilization of an Arrhenius model for maize kernels treated using different drying conditions.

For the kernels treated using HAVD 55 °C and HAVD 65 °C, the values of *α_T_* have the potential to be the same at 40–45 °C for both *E*′ and *E*″. This suggests that, at 40–45 °C, both the kernels treated with HAVD 55 °C and HAVD 65 °C behave like thermorheologically simple materials. The 30 °C ITF data for *E*′ and *E*″ can superpose the 35 °C ITF data for *E*′ and *E*″ in the meantime, while the 35 °C ITF data for *E*′ and *E*″ cannot superpose the 40 °C ITF data for *E*′ and *E*″ simultaneously. Therefore, the master curves for *E*′ and *E*″ cannot be generated using exactly the same values of *α_T_* for the kernels treated by HAVD 55 °C and HAVD 65 °C. They were generated using different values of *α_T_* (Table 2) and plotted separately (Figure 2(D_2_(*E*′),D_2_′(*E*″),E_2_(*E*′),E_2_′(*E*″))). A maize kernel is a polymer and contains starch, lipids, proteins, and other components. Temperature dependence is different for every component. The heterogeneous nature of maize kernel might account for this lack of superposition. To address this issue, the vertical shift factor *b_T_* might be required. Nevertheless, all the curves (Figure 2(D_2_(*E*′),D_2_′(*E*″),E_2_(*E*′),E_2_′(*E*″))) show superposition. Zhu et al. [24] applied TTS to predict the highland barley kernels’ creep properties. TTS was applicable at low temperatures. Nevertheless, TTS did not hold when temperature surpassed a certain value. Meza et al. [11] compared the refrigerated and frozen cheeses for the FTS, reporting that the values of *α_T_* were identical at 10–30 °C for both loss modulus and storage modulus. However, the *α_T_* values were not the same at 40–50 °C for both loss modulus and storage modulus.

For the kernels treated using ND, HAVD 35 °C, HAVD 45 °C, and HAVD 75 °C, the values of *α_T_* are identical at 30–45 °C for both *E*′ and *E*″. That is, the master curves for *E*′ and *E*″ can be generated using exactly the same values of *α_T_*. The master curves for *E*′ and *E*″ were plotted in the same figure (Figure 2(A_2_,B_2_,C_2_,F_2_)) for all these samples. All these curves (Figure 2(A_2_,B_2_,C_2_,F_2_)) show superposition for the FTS, despite having a number of tails and irregularities. These tails and irregularities are related to the relaxation mechanism, which occurs inside the maize kernel and is dependent on temperature [25]. Nevertheless, in general, the master curves can still be considered continuous and smooth. Both *E*′ and *E*″ of the kernels treated using HAVD 45 °C and HAVD 75 °C (Figure 2(C_2_,F_2_)) exhibit excellent fit for the FTS. This suggests that the maize kernels treated with HAVD 45 °C and HAVD 75 °C show thermorheologically simple behavior. Furthermore, the values of *α_T_* for HAVD 35 °C and HAVD 75 °C are relatively higher (at the same temperature), implying higher temperature sensitivity. This higher value of *α_T_* also implies that the prediction for *E*′ and *E*″ covers a wider range of frequency. Overall, the frequency range was extended from 1 to 4 log periods using FTS. This range was far beyond the instrument’s measurement frequency range. It is extremely challenging to conduct a DMA test for such a high frequency. The success of FTS indicates that very little variations take place inside the maize kernels treated using HAVD 45 °C and HAVD 75 °C in the range of 30–45 °C, and the moduli show comparable temperature dependence [23]. In principle, FTS will not hold for inhomogeneous polymeric material, as the temperature dependences of components of material are different. However, FTS holds for the maize kernels treated with HAVD 45 °C and HAVD 75 °C. This may be because the proportion of carbohydrates in maize kernels is as high as 75%, while the proportion of other components is small. Thus, carbohydrates primarily contribute to the measured stress signal, while other components’ contribution to the measured stress signal is relatively small [26]. FTS has also been applied to guar gum dispersions [25]. In that study, the frequency scale of guar gum dispersions was extended from 2 to 4–5 log periods.

As shown in Table 2, as the temperature decreases, *α_T_* increases monotonically. Similar results were reported in previous studies [11,27]. As shown in Table 3, the Arrhenius model is capable of describing the temperature dependence of *α_T_* (*R*^2^ ≥ 0.844). Nevertheless, for some samples, the *R*^2^ was not very high. This occurs on account of the closeness of the temperatures; hence, no obvious rheological changes were detected. A similar result was reported in a previous study [25].

Since the master curves for *E*′ and *E*″ were generated using different values of *α_T_* for the kernels treated by HAVD 55 °C and HAVD 65 °C, the *E_a_* for *E*′ and *E*″ were also different (Table 3). As shown in Table 3, *E_a_* ranges from 191.014 to 457.004 kJ/mol, which is greater than the stress relaxation activation energy (90.86–134.8 kJ/mol) for maize kernel [28]. The kernel treated using HAVD 65 °C demonstrates the highest *E_a_*. This indicates that greater energy is needed for polymeric chain rearrangement of the kernel treated with HAVD 65 °C. The kernel treated by HAVD 55 °C has the lowest *E_a_*. Meza et al. [11] acquired an activation energy of 165.7 ± 15.6 kJ/mol for frozen cheese and 158.1 ± 8.8 kJ/mol for refrigerated cheese.

### 3.3. Modified Cole–Cole Plot

**Figure 3:**
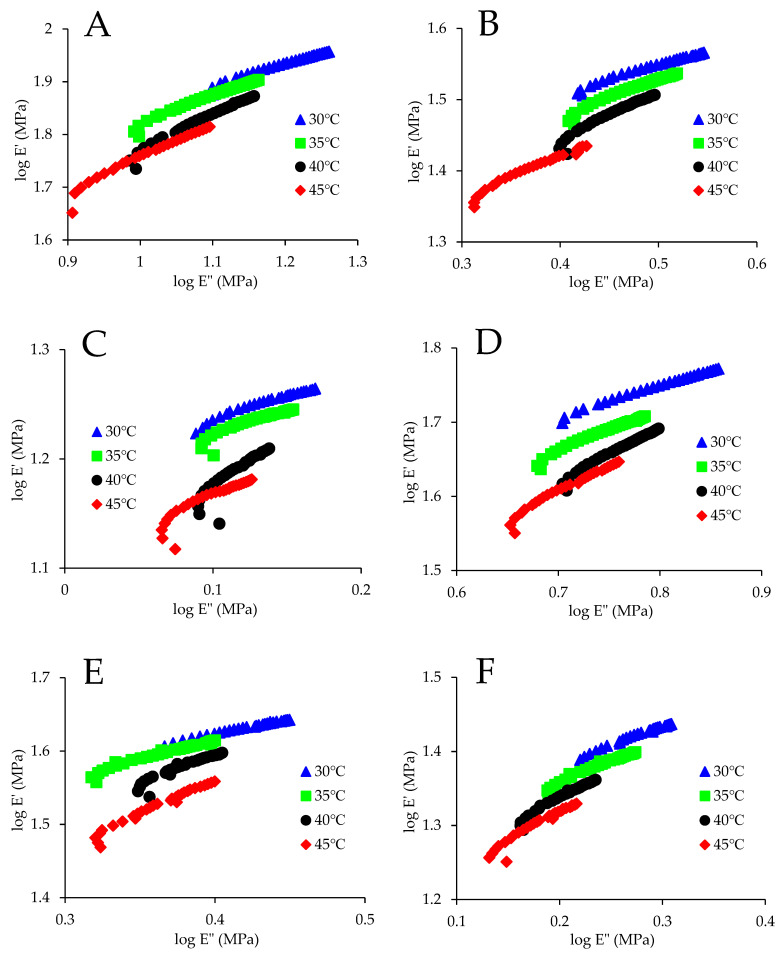
Modified Cole–Cole plot for maize kernels treated using different drying conditions. (**A**)—ND; (**B**)—HAVD 35 °C; (**C**)—HAVD 45 °C; (**D**)—HAVD 55 °C; (**E**)—HAVD 65 °C; (**F**)—HAVD 75 °C.

Figure 3 shows the MCC plot for maize kernels treated using different drying conditions. For the kernel treated by HAVD 75 °C (Figure 3F), ITF curves superposed in the whole temperature range (30–45 °C). This suggests that the kernel treated by HAVD 75 °C exhibited thermorheologically simple behavior in the whole temperature range (30–45 °C). This is in accordance with the result obtained by applying FTS. Furthermore, curvature was observed in Figure 3F. Similar phenomena were reported by a previous study [29]. The MCC plot would be linear in completely miscible systems [30]. The heterogeneous nature of maize kernel might account for the curvature visible in Figure 3F.

We found that MCC analysis was more sensitive as compared with FTS. For example, for the kernel treated by HAVD 45 °C, the ITF curves superposed in the whole temperature range (30–45 °C) using FTS, while the 35 °C ITF curve failed to superpose the 40 °C ITF curve using the MCC analysis (Figure 3C). This finding is consistent with the finding of Han and Kim [30]. They considered TTS (FTS) less sensitive than the MCC analysis because the MCC analysis is sensitive to microstructural heterogeneity within the material. Overall, both MCC analysis and FTS succeeded in the kernel treated by HAVD 75 °C.

### 3.4. Wider Frequency Range

**Figure 4:**
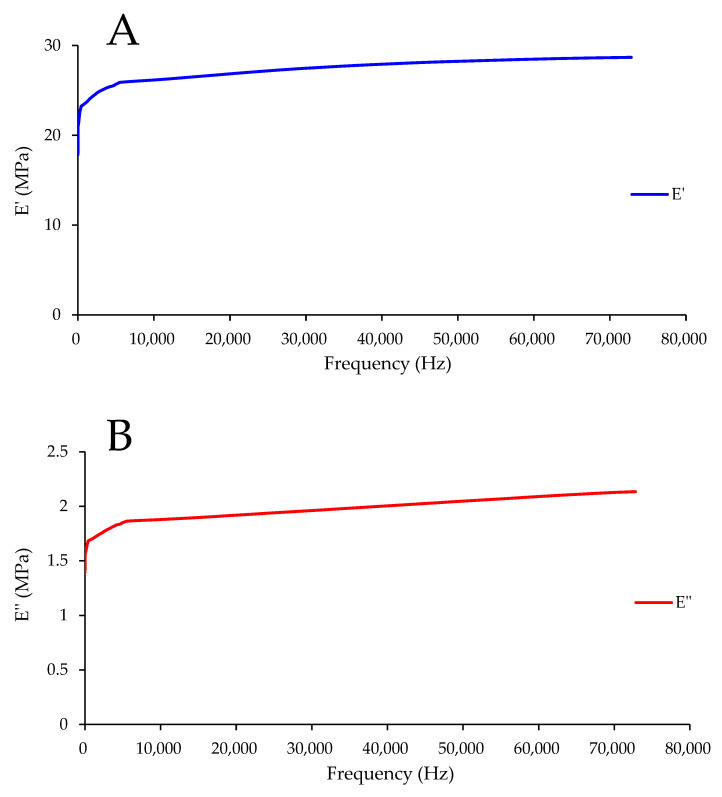
Frequency–temperature superposition predicted values of *E*′ and *E*″ for the maize kernel treated by HAVD 75 °C. (**A**)—*E*′; (**B**)—*E*″.

The values of *E*′ and *E*″ predicted using FTS for the maize kernel treated with HAVD 75 °C at 45 °C are displayed in Figure 4. The obtained predicted curves provide a characterization up to 70,000 Hz. The predicted values of *E*′ and *E*″ were fitted to the power law equations (Equations (1) and (2)), and the obtained parameters are displayed in Table 4. The *R*^2^ was 0.979 for *E*″ and 0.995 for *E*′. This indicates that the power law equation can fit the *E*′ and *E*″ data well. *K*′ is higher than *K*″. This is in line with the result visible in Figure 4. *n*′ is somewhat higher than *n*″, suggesting that the frequency dependence of *E*′ is moderately higher than that of *E*″.

The predicted values of *E*′ and *E*″ were also fitted to the generalized Maxwell model:

(4) $$E^{\prime }=E_{e}+\sum_{i=1}^{n}E_{i}\frac{{\left(\lambda_{i}\omega \right)}^{2}}{{\left(\lambda_{i}\omega \right)}^{2}+1}$$

(5) $$E\prime \prime =\sum_{i=1}^{n}E_{i}\frac{\lambda_{i}\omega }{{\left(\lambda_{i}\omega \right)}^{2}+1}$$

where *E_e_* (MPa) denotes the equilibrium modulus (*E_e_* is zero for liquids (*E_e_* = 0) and finite for solids (*E_e_* > 0)), *ω* (rad/s) denotes the angular frequency, *n* denotes the number of relaxation modes, and *λ_i_* (s) and *E_i_* (MPa) denote the *i*th relaxation time and *i*th relaxation strength, respectively. TA Instruments Trios software (version 5.1.1, New Castle, DE, USA) was utilized to compute the generalized Maxwell model parameters.

The generalized Maxwell model parameters are listed in Table 5. The value of *E_e_* was 15.998 MPa. The *R*^2^ was 0.989, suggesting the generalized Maxwell model can fit the FTS predicted values of *E*′ and *E*″ well. As shown in Table 5, most of the *λ_i_* values were detected for fast dynamics relaxation processes. Fast dynamics is related to small-scale relaxation processes (subunits of molecules or molecular strands) [31]. Thus, in this study, we can assert that the relaxation processes in the kernel treated with HAVD 75 °C are mainly related to molecular strands or subunits. A similar result was reported in a previous study [32].

## 4. Conclusions

We performed frequency sweep experiments, FTS, and MCC analysis on the maize kernels treated by ND and HAVD. The maize kernels’ viscoelastic properties for HAVD were higher than those for ND. The results of the frequency sweep experiments can provide insights into the viscoelastic behavior of maize kernel under the vibration of production line or transportation vehicles. This can provide theoretical guidance for reducing maize kernel damage in processing or transportation. The frequency range for the kernel treated using HAVD 75 °C extended up to 70,000 Hz. This can be utilized to estimate the texture of maize kernel at ultra-high frequencies. The heterogeneous nature of maize kernel might account for the curvature in MCC plot and the failure of FTS. MCC analysis was more sensitive than FTS. The relaxation processes in the kernel treated by HAVD 75 °C are mainly associated with molecular strands or subunits. Future work should incorporate small-angle X-ray scattering measurements to characterize the structure of maize kernel treated using HAVD. The industry can utilize the data of this investigation to calculate the texture attributes when developing their maize products for increasing the product quality.

## Figures and Tables

**Table 1:** Power law model parameters for maize kernels dried utilizing various drying treatments.

Drying Treatment	Sample Size (Thickness × *D* *)	*T* (°C)	*E*′ *= K*′*·f^n^*^′^			*E*″ *= K*″*·f^n”^*		
			*n*′	*K*′ (MPa·s^n^)	*R* ^2^	*n*″	*K*″ (MPa·s^n^)	*R* ^2^
ND	3.848 mm × 8.789 mm	30	0.064	72.476	0.979	0.148	10.709	0.927
	3.848 mm × 8.789 mm	35	0.083	60.005	0.982	0.152	8.422	0.926
	3.848 mm × 8.789 mm	40	0.103	51.683	0.977	0.146	8.389	0.917
	3.848 mm × 8.789 mm	45	0.121	42.506	0.984	0.174	6.609	0.917
HAVD 35 °C	4.073 mm × 8.755 mm	30	0.049	31.068	0.969	0.115	2.330	0.916
	4.073 mm × 8.755 mm	35	0.056	28.296	0.976	0.098	2.318	0.901
	4.073 mm × 8.755 mm	40	0.065	25.627	0.980	0.084	2.304	0.891
	4.073 mm × 8.755 mm	45	0.066	21.432	0.964	0.101	1.831	0.874
HAVD 45 °C	4.388 mm × 9.144 mm	30	0.035	16.331	0.994	0.069	1.147	0.905
	4.388 mm × 9.144 mm	35	0.030	15.901	0.996	0.052	1.177	0.874
	4.388 mm × 9.144 mm	40	0.051	13.598	0.992	0.038	1.186	0.769
	4.388 mm × 9.144 mm	45	0.045	13.036	0.996	0.052	1.103	0.843
HAVD 55 °C	4.588 mm × 9.313 mm	30	0.056	48.636	0.980	0.132	4.504	0.951
	4.588 mm × 9.313 mm	35	0.055	42.159	0.984	0.092	4.382	0.924
	4.588 mm × 9.313 mm	40	0.063	39.313	0.977	0.080	4.700	0.915
	4.588 mm × 9.313 mm	45	0.071	34.325	0.972	0.090	4.100	0.886
HAVD 65 °C	4.647 mm × 9.117 mm	30	0.030	39.577	0.990	0.070	2.195	0.956
	4.647 mm × 9.117 mm	35	0.043	35.440	0.981	0.072	1.934	0.890
	4.647 mm × 9.117 mm	40	0.045	33.877	0.985	0.046	2.131	0.832
	4.647 mm × 9.117 mm	45	0.070	28.119	0.963	0.066	1.952	0.876
HAVD 75 °C	3.979 mm × 8.845 mm	30	0.042	23.725	0.976	0.078	1.549	0.940
	3.979 mm × 8.845 mm	35	0.045	21.469	0.968	0.076	1.432	0.919
	3.979 mm × 8.845 mm	40	0.054	19.106	0.978	0.065	1.362	0.908
	3.979 mm × 8.845 mm	45	0.063	17.147	0.972	0.070	1.271	0.853

* *D* was the geometric mean of kernel width and kernel length.

**Table 2:** Horizontal shift factor *α_T_* for maize kernels dried utilizing various drying treatments.

Drying Treatment	*T* (°C)	log*α_T_* (*E*′)	log*α_T_* (*E*″)
ND	30	2.868	2.868
	35	1.628	1.628
	40	1.004	1.004
	45	0.000	0.000
HAVD 35 °C	30	3.217	3.217
	35	2.358	2.358
	40	1.457	1.457
	45	0.000	0.000
HAVD 45 °C	30	2.717	2.717
	35	1.997	1.997
	40	0.915	0.915
	45	0.000	0.000
HAVD 55 °C	30	3.503	1.722
	35	2.087	1.195
	40	1.121	1.168
	45	0.000	0.000
HAVD 65 °C	30	3.795	2.171
	35	2.338	1.278
	40	1.351	1.174
	45	0.000	0.000
HAVD 75 °C	30	3.385	3.385
	35	2.264	2.264
	40	1.147	1.147
	45	0.000	0.000

**Table 3:** Activation energy (*E_a_*) obtained utilizing Arrhenius model for maize kernels dried utilizing various drying treatments.

Drying Treatment	*E_a_* (kJ/mol)	*R* ^2^
ND	341.049	0.987
HAVD 35 °C	388.955	0.978
HAVD 45 °C	340.723	0.993
HAVD 55 °C	424.109 (for *E*′), 191.014 (for *E*″)	0.995 (for *E*′), 0.844 (for *E*″)
HAVD 65 °C	457.004 (for *E*′), 244.030 (for *E*″)	0.996 (for *E*′), 0.917 (for *E*″)
HAVD 75 °C	416.221	0.999

**Table 4:** Power law parameters for the maize kernel treated by HAVD 75 °C.

Drying Treatment	*E*′ *= K*′*·f^n^*^′^			*E*″ *= K*″*·f^n^*^″^		
	*n*′	*K*′ (MPa·s^n^)	*R* ^2^	*n*″	*K*″ (MPa·s^n^)	*R* ^2^
HAVD 75 °C	0.042	17.871	0.995	0.038	1.338	0.979

**Table 5:** Generalized Maxwell model parameters for the maize kernel treated by HAVD 75 °C.

*x*	*λ_i_* (s)	*E_i_* (MPa)
1	2.186 × 10^−6^	3.473
2	1.544 × 10^−5^	1.733
3	4.590 × 10^−5^	1.125
4	1.237 × 10^−4^	1.033
5	4.065 × 10^−4^	0.579
6	4.090 × 10^−4^	0.894
7	1.536 × 10^−3^	1.257
8	6.213 × 10^−3^	1.509
9	0.026	1.240
10	0.159	2.248

## Data Availability

The original contributions presented in the study are included in the article, further inquiries can be directed to the corresponding author.
